# The Genetic Structure of Marijuana and Hemp

**DOI:** 10.1371/journal.pone.0133292

**Published:** 2015-08-26

**Authors:** Jason Sawler, Jake M. Stout, Kyle M. Gardner, Darryl Hudson, John Vidmar, Laura Butler, Jonathan E. Page, Sean Myles

**Affiliations:** 1 Department of Plant and Animal Sciences, Faculty of Agriculture, Dalhousie University, Truro, Nova Scotia, B2N 5E3, Canada; 2 Anandia Labs, 2259 Lower Mall, Vancouver, British Columbia, V6T 1Z4, Canada; 3 Department of Biological Sciences, University of Manitoba, Winnipeg, Manitoba, R3T 2N2, Canada; 4 The DOC Solutions, 213 West 32nd St, Hamilton, Ontario, L9C 5H3, Canada; 5 Alberta Innovates-Technology Futures, P.O. Bag 4000, Vegreville, Alberta, T9C 1T4, Canada; 6 Botany Department, University of British Columbia, #3529–6270 University Blvd, Vancouver, British Columbia, V6T 1Z4, Canada; Agriculture and Agri-Food Canada, CANADA

## Abstract

Despite its cultivation as a source of food, fibre and medicine, and its global status as the most used illicit drug, the genus *Cannabis* has an inconclusive taxonomic organization and evolutionary history. Drug types of *Cannabis* (marijuana), which contain high amounts of the psychoactive cannabinoid **Δ** ^9^-tetrahydrocannabinol (THC), are used for medical purposes and as a recreational drug. Hemp types are grown for the production of seed and fibre, and contain low amounts of THC. Two species or gene pools (*C*. *sativa* and *C*. *indica*) are widely used in describing the pedigree or appearance of cultivated *Cannabis* plants. Using 14,031 single-nucleotide polymorphisms (SNPs) genotyped in 81 marijuana and 43 hemp samples, we show that marijuana and hemp are significantly differentiated at a genome-wide level, demonstrating that the distinction between these populations is not limited to genes underlying THC production. We find a moderate correlation between the genetic structure of marijuana strains and their reported *C*. *sativa* and *C*. *indica* ancestry and show that marijuana strain names often do not reflect a meaningful genetic identity. We also provide evidence that hemp is genetically more similar to *C*. *indica* type marijuana than to *C*. *sativa* strains.

## Introduction

*Cannabis* is one of humanity’s oldest crops, with records of use dating to 6000 years before present. Possibly because of its early origins, and due to restrictions on scientific inquiry brought about by drug policy, the evolutionary and domestication history of *Cannabis* remains poorly understood. Hillig (2005) proposed on the basis of allozyme variation that the genus consists of three species (*C*. *sativa*, *C*. *indica*, and *C*. *ruderalis*) [1], whereas an alternative viewpoint is that *Cannabis* is monotypic and that observable subpopulations represent subspecies of *C*. *sativa* [2]. The putative species *C*. *ruderalis* may represent feral populations of the other types or those adapted to northern regions.

The classification of *Cannabis* populations is confounded by many cultural factors, and tracing the history of a plant that has seen wide geographic dispersal and artificial selection by humans over thousands of years has proven difficult. Many hemp types have varietal names while marijuana types lack an organized horticultural registration system and are referred to as strains. The draft genome and transcriptome of *C*. *sativa* were published in 2011 [3], however until now there has been no published investigation of *Cannabis* population structure using high-throughput genotyping methods. As both public opinion and legislation in many countries shifts towards recognizing *Cannabis* as a plant of medical and agricultural value [4], the genetic characterization of marijuana and hemp becomes increasingly important for both clinical research and crop improvement efforts.

An important first step towards deeper evolutionary and functional analyses of *Cannabis*, including trait mapping and identification of functional genetic variation, is the characterization of the genetic structure of the genus. Here, we report the genotyping of a diverse collection of *Cannabis* germplasm and show that genetic differences between hemp and marijuana are not limited to genes involved in THC production, while the reported *C*. *sativa* and *C*. *indica* ancestries of marijuana strains only partially capture the main axes of marijuana’s genetic structure.

## Results and Discussion

**Fig 1:**
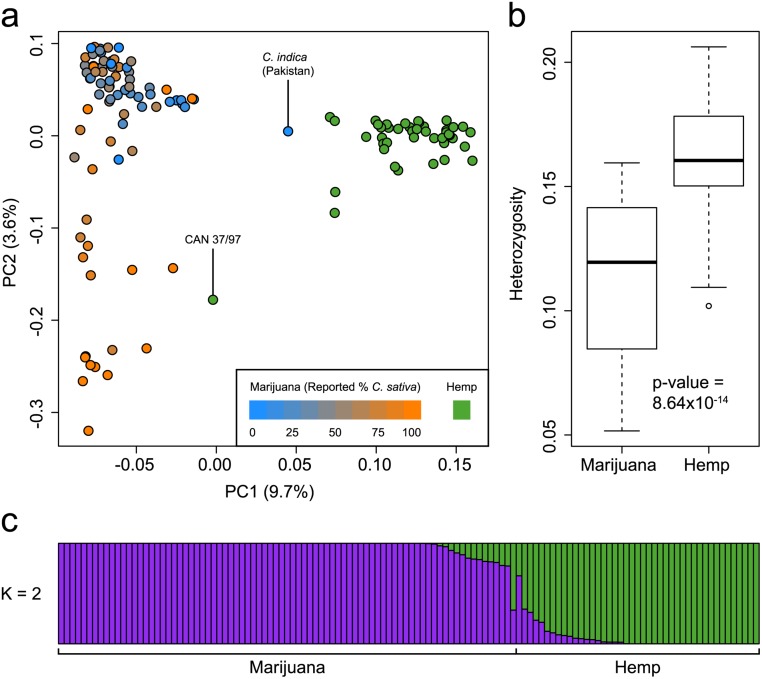
Genetic structure of marijuana and hemp. (a) PCA of 43 hemp and 81 marijuana samples using 14,031 SNPs. Hemp samples are colored green and marijuana samples are colored according to their reported *C*. *sativa* ancestry. The proportion of the variance explained by each PC is shown in parentheses along each axis. The two samples labeled with their IDs are discussed in the text. (b) Boxplots showing significantly lower heterozygosity in marijuana than in hemp. (c) Population structure of hemp and marijuana estimated using the fastSTRUCTURE admixture model at K = 2. Each sample is represented by a thin vertical line, which is partitioned into two colored segments that represent the sample’s estimated membership in each of the two inferred clusters. Hemp and marijuana samples are labeled below the plot.

To evaluate the genetic structure of commonly cultivated *Cannabis*, we genotyped 81 marijuana and 43 hemp samples using genotyping-by-sequencing (GBS) [5]. The marijuana samples represent a broad cross section of modern commercial strains and landraces, while the hemp samples include diverse European and Asian accessions and modern varieties. Although we sampled a diverse collection of cannabis types in our study, access to samples is complicated by the fact that marijuana is an illicit drug and there are limited repositories of hemp germplasm in international seedbanks. In total, 14,031 SNPs were identified after applying quality and missingness filters. Principal components analysis (PCA) of both marijuana and hemp (Fig 1a) revealed clear genetic structure separating marijuana and hemp along the first principal component (PC1). This distinction was further supported using the fastSTRUCTURE algorithm [6] assuming K = 2 ancestral populations (Fig 1c). PCA and fastSTRUCTURE produced highly similar results: a sample’s position along PC1 was strongly correlated with its group membership according to fastSTRUCTURE at K = 2 (r^2^ = 0.98; p-value < 1 x 10^−15^).

We observe a putative *C*. *indica* marijuana strain from Pakistan that is genetically more similar to hemp than it is to other marijuana strains (Fig 1a). Similarly, the hemp sample CAN 37/97 clusters more closely with marijuana strains (Fig 1a). These outliers may be due to sample mix-up or their classification as hemp or marijuana may be incorrect. The sample of CAN 37/97 that we genotyped was from a Canadian hemp germplasm collection, which obtained this accession from the IPK Genebank (Gatersleben, Germany). The original source country is France but there is limited information to indicate the cultivation of CAN 37/97 as hemp. Alternatively, these samples may be true outliers and represent exceptional strains that are genetically unlike others in their group. Using the current data set, the unambiguous identification of a sample as either hemp or marijuana would be possible in the former case, but not in the latter. In any case, we find that the primary axis of genetic variation in *Cannabis* differentiates hemp from marijuana.

These results significantly expand our understanding of the evolution of marijuana and hemp lineages in *Cannabis*. Previous analyses have shown that marijuana and hemp differ in their capacity for cannabinoid biosynthesis, with marijuana possessing the *B*_T_ allele coding for tetrahydrocannabinolic acid synthase and hemp typically possessing the *B*_D_ allele for cannabidiolic acid synthase [7]. As well, transcriptome analysis of female flowers showed that cannabinoid pathway genes are significantly upregulated in marijuana compared to hemp, as expected from the very high THC levels in the former compared to the latter [3]. Our results indicate that the genetic differences between the two are distributed across the genome and are not restricted to loci involved in cannabinoid production. In addition, we find that levels of heterozygosity are higher in hemp than in marijuana (Fig 1b; Mann-Whitney U-test, p-value = 8.64 x 10^−14^), which suggests that hemp cultivars are derived from a broader genetic base than that of marijuana strains and/or that breeding among close relatives is more common in marijuana than in hemp.

The difference between marijuana and hemp plants has considerable legal implications in many countries, and to date forensic applications have largely focused on determining whether a plant should be classified as drug or non-drug [8]. EU and Canadian regulations only permit hemp cultivars containing less than 0.3% THC to be grown. While hemp and marijuana appear relatively well separated along PC1 (Fig 1a), we found no SNPs with fixed differences between these two groups: the highest F_ST_ value between hemp and marijuana among all 14,031 SNPs was 0.87 for a SNP with an allele frequency of 0.82 in hemp and 0 in marijuana (S1 Table). The average F_ST_ between hemp and marijuana is 0.156 (S1 Fig), which is similar to the degree of genetic differentiation in humans between Europeans and East Asians [9]. Thus, while cannabis breeding has resulted in a clear genetic differentiation according to use, hemp and marijuana still largely share a common pool of genetic variation.

**Fig 2:**
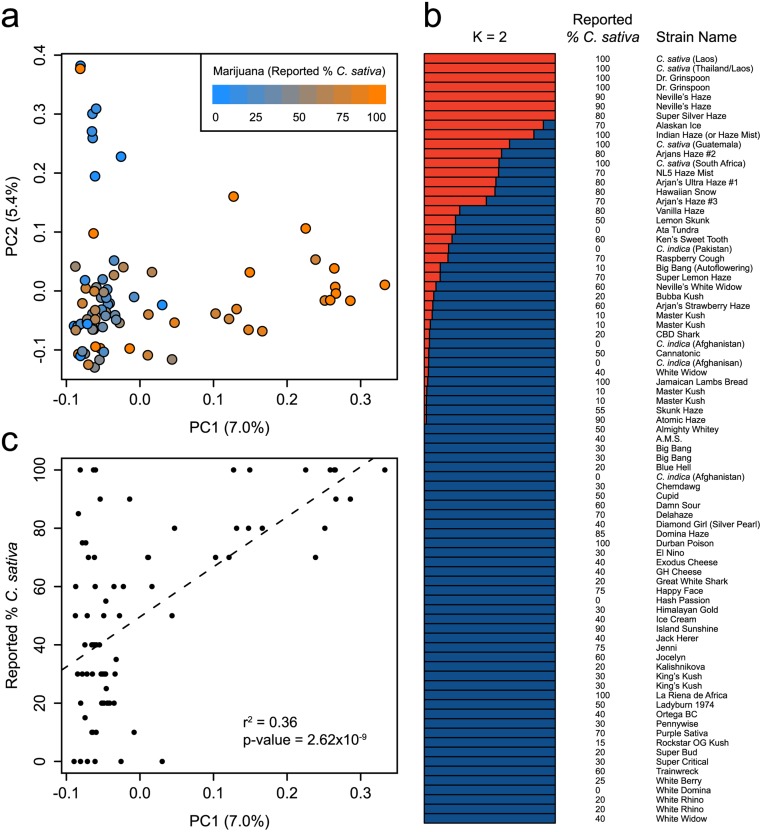
Genetic structure of marijuana. (a) PCA of 81 marijuana samples using 9,776 SNPs. Samples are colored according to their reported *C*. *sativa* ancestry. The proportion of the variance explained by each PC is shown in parentheses along each axis. (b) Population structure of marijuana calculated using the fastSTRUCTURE admixture model at K = 2. Each sample is represented by a horizontal bar, which is partitioned into two colored segments that represent the sample’s estimated membership in each of the two inferred clusters. Adjacent to each bar is the sample’s name and reported % *C*. *sativa* ancestry. (c) The correlation between the principal axis of genetic structure (PC1) in marijuana and reported *C*. *sativa* ancestry.

Although the taxonomic separation of the putative taxa *C*. *sativa* and *C*. *indica* remains controversial, a vernacular taxonomy that distinguishes between “Sativa” and “Indica” strains is widespread in the marijuana community. Sativa-type plants, tall with narrow leaves, are widely believed to produce marijuana with a stimulating, cerebral psychoactive effect while Indica-type plants, short with wide leaves, are reported to produce marijuana that is sedative and relaxing. We find that the genetic structure of marijuana is in partial agreement with strain-specific ancestry estimates obtained from various online sources (Fig 2, S2 Table). We observe a moderate correlation between the positions of marijuana strains along the first principal component (PC1) of Fig 2a and reported estimates of *C*. *sativa* ancestry (Fig 2c)(r^2^ = 0.36; p-value = 2.62 x 10^−9^). This relationship is also observed for the second principal component (PC2) of Fig 1a (r^2^ = 0.34; p-value = 1.21 x 10^−8^). This observation suggests that *C*. *sativa* and *C*. *indica* may represent distinguishable pools of genetic diversity [1] but that breeding has resulted in considerable admixture between the two. While there appears to be a genetic basis for the reported ancestry of many marijuana strains, in some cases the assignment of ancestry strongly disagrees with our genotype data. For example we found that Jamaican Lambs Bread (100% reported *C*. *sativa*) was nearly identical (IBS = 0.98) to a reported 100% *C*. *indica* strain from Afghanistan. Sample mix-up cannot be excluded as a potential reason for these discrepancies, but a similar level of misclassification was found in strains obtained from Dutch coffee shops based on chemical composition [10]. The inaccuracy of reported ancestry in marijuana likely stems from the predominantly clandestine nature of *Cannabis* growing and breeding over the past century. Recognizing this, marijuana strains sold for medical use are often referred to as Sativa or Indica “dominant” to describe their morphological characteristics and therapeutic effects [10]. Our results suggest that the reported ancestry of some of the most common marijuana strains only partially captures their true ancestry.

As a wind-pollinated dioecious plant (though monoecious forms exist), *Cannabis* is highly heterozygous and many marijuana strains are clonally propagated in order to retain their genetic identity. In contrast to other clonally propagated crops like apples and grapes, however, strain names are assigned to marijuana plants even if grown from seed. Thus, a marijuana strain name does not necessarily represent a genetically unique variety. To investigate the genetic identity of named marijuana strains at the genetic level, we compared samples with identical names to each other and to all other genotyped samples. We found that in 6 of 17 comparisons (35%), samples were more genetically similar to samples with different names than to samples with identical names. We conclude that the genetic identity of a marijuana strain cannot be reliably inferred by its name or by its reported ancestry.

Hemp is consistently classified as *C*. *sativa* in previously published literature [11, 12], and the prevailing assumption has been that varieties used for fibre and seed production are derived from *C*. *sativa* [1]. Our results are incompatible with this proposition: a marijuana strain’s genetic distance to hemp is negatively correlated with its reported *C*. *sativa* ancestry (r^2^ = 0.17; p-value = 0.0002) and is negatively correlated with its position along PC1 of Fig 2a (r^2^ = 0.43; p-value = 4.63 x 10^−11^). Moreover, we find that F_ST_ is higher between hemp and marijuana strains with 100% reported *C*. *sativa* ancestry (F_ST_ = 0.161) than between hemp and strains with 100% reported *C*. *indica* ancestry (F_ST_ = 0.136). Hillig (2005) challenged the *C*. *sativa* origin of hemp, and noted that fibre/seed strains from Asia cluster with *C*. *indica* [1]. Similarly, a recent study using Random Amplified Polymorphic DNA (RAPD) markers found that *C*. *indica* clustered more closely with hemp than with *C*. *sativa* or hybrid marijuana strains [8]. Consistent with these studies, our findings suggest that hemp has a greater proportion of alleles in common with *C*. *indica* than with *C*. *sativa*.

There is a paucity of public repositories for hemp germplasm and only a patchwork of private collections of marijuana strains worldwide. These factors, and the low viability of *Cannabis* seed after prolonged storage, cause concern that the genomic variation that we describe here is in danger of being lost. After decades of restrictive regulations and the replacement of hemp fibre with synthetic products, *Cannabis* cultivation is now undergoing a resurgence in many parts of the world. For example, Canadian hemp acreage reached 27,000 ha in 2013 [13] and support for hemp research was included in the recent US Farm Bill [14]. The present study provides clarity on the genetic structure of marijuana and hemp and highlights the severe challenges associated with marijuana germplasm curation due to its clandestine past. Achieving a practical, accurate and reliable classification system for *Cannabis*, including a variety registration system for marijuana-type plants, will require significant scientific investment and a legal framework that accepts both licit and illicit forms of this plant. Such a system is essential in order to realize the enormous potential of *Cannabis* as a multi-use crop (hemp) and as a medicinal plant (marijuana).

## Materials and Methods

### Genetic material and genotyping

The marijuana strains genotyped in this study were provided by author DH (grown by Health Canada authorized producers) and represent germplasm grown and used for breeding in the medical and recreational marijuana industries (S2 Table). Hemp strains were provided by author JV (Health Canada hemp cultivation licensee), and represent modern seed and fibre cultivars grown in Canada as well as diverse European and Asian germplasm (S3 Table). DNA was extracted from hemp leaf tissue using a Qiagen DNeasy plant mini kit, and from marijuana leaves using a Macherey-Nagel NucleoSpin 96 Plant II kit with vacuum manifold processing. Library preparation and sequencing were performed using the GBS protocol published by Sonah et al [15]. The raw sequence has been deposited in the NIH Sequence Read Archive (SRA), under BioProject PRJNA285813. SNPs with a read depth of 10 or more were called using the GBS pipeline developed by Gardner et al. [16], aligning to the canSat3 *C*. *sativa* reference genome assembly [3]. Quality filtering of genetic markers was performed in PLINK 1.07 [17] by removing SNPs with (i) greater than 20% missingness by locus (ii) a minor allele frequency less than 1% and (iii) excess heterozygosity (a Hardy-Weinberg equilibrium p-value less than 0.0001). After filtering, 14,031 SNPs remained for analysis.

### Collection of reported marijuana ancestry

Reported ancestry proportions (% *C*. *sativa* and % *C*. *indica*) were manually obtained from online strain databases, *Cannabis* seed retailers, and licensed producers of medical marijuana (S2 Table). Author DH provided ancestry estimates for 26 strains for which no online information was available.

### Analysis of population structure and heterozygosity

Principal components analysis (PCA) was performed using PLINK 1.9 (www.cog-genomics.org/plink2)[18]. fastSTRUCTURE [6] was run at K = 2 and K = 3 using default parameters for hemp and marijuana samples combined (14,031 SNPs) (Fig 1a and 1c), and marijuana samples alone (9,776 SNPs) (Fig 2a and 2b). The analysis at K = 2 was performed to test the extent to which the samples reflect two distinct groups. Other values of K were tested (not shown), but did not provide further optimization or descriptive value. Heterozygosity by individual was calculated in R by dividing the number of heterozygous sites by the number of non-missing genotypes for each sample. As a result of the genotyping method used in this study, we anticipate the systematic miscalling of heterozyote genotypes as homozygous due to our sequencing depth threshold of ≥10 reads (See Fig 2 in [19]).

### Identity by state (IBS) analysis

Pairwise proportion IBS between all pairs of samples was calculated using PLINK 1.07. One outlier was excluded from this analysis, *C*. *indica* (Pakistan), because of its significantly higher IBS to hemp than all other marijuana strains (Labeled marijuana sample in Fig 1a).

To determine if the hemp population shared greater allelic similarity to *C*. *sativa* or *C*. *indica* marijuana, we calculated the mean pairwise IBS between each marijuana strain and all hemp strains. We performed this analysis at various minor allele frequency thresholds and the result remained unchanged.

## Supporting Information

S1 Fig Distribution of F_ST_ between marijuana and hemp samples across 14,031 SNPs. (a) F_ST_ distribution for all SNPs genotyped. (b) Distribution of SNPs with F_ST_ greater than 0.5. Average F_ST_ is weighted by allele frequency and was calculated according to equation 10 in Weir and Cockerham (1984) [20].
(PDF)

S2 Fig Mean pairwise IBS between each marijuana sample and all hemp samples versus reported *C*. *sativa* ancestry. (PDF)

S1 Table Positions and allele frequencies of the top 50 SNPs by F_ST_ between marijuana and hemp calculated according to equation 10 in Weir and Cockerham (1984) [20]. (XLSX)

S2 Table Sample names and reported *C*. *sativa* and *C*. *indica* ancestry of genotyped marijuana strains. (XLSX)

S3 Table Sample names of genotyped hemp varieties. (XLSX)

S4 Table Keyfiles containing barcodes and additional metadata used in the GBS pipeline. (XLSX)
