# Supplementary figures and images for: The Genetic Structure of Marijuana and Hemp
Source: PLoS One. 2015 Aug 26;10(8):e0133292. doi: 10.1371/journal.pone.0133292 (PMC4550350; doi:10.1371/journal.pone.0133292)

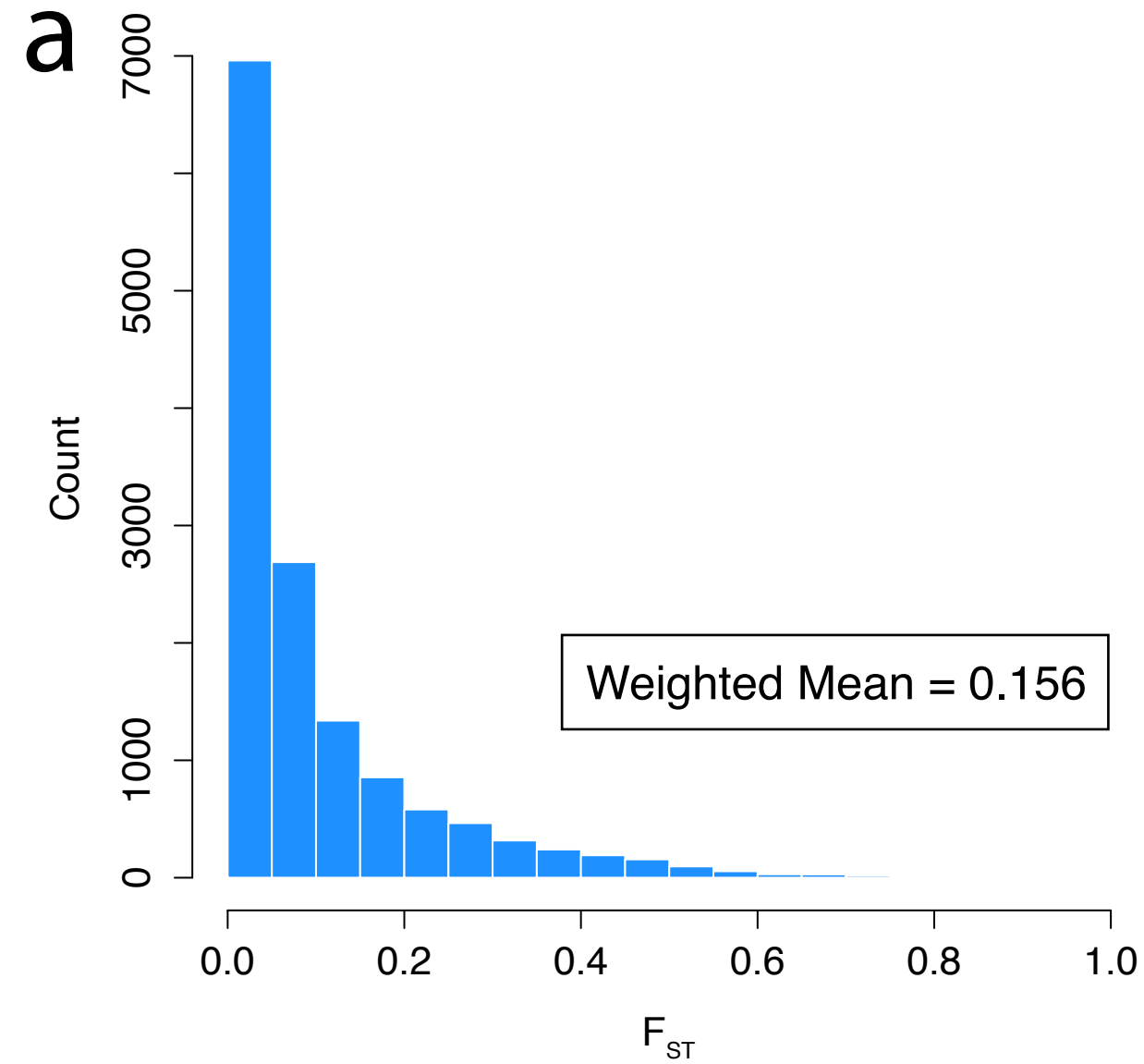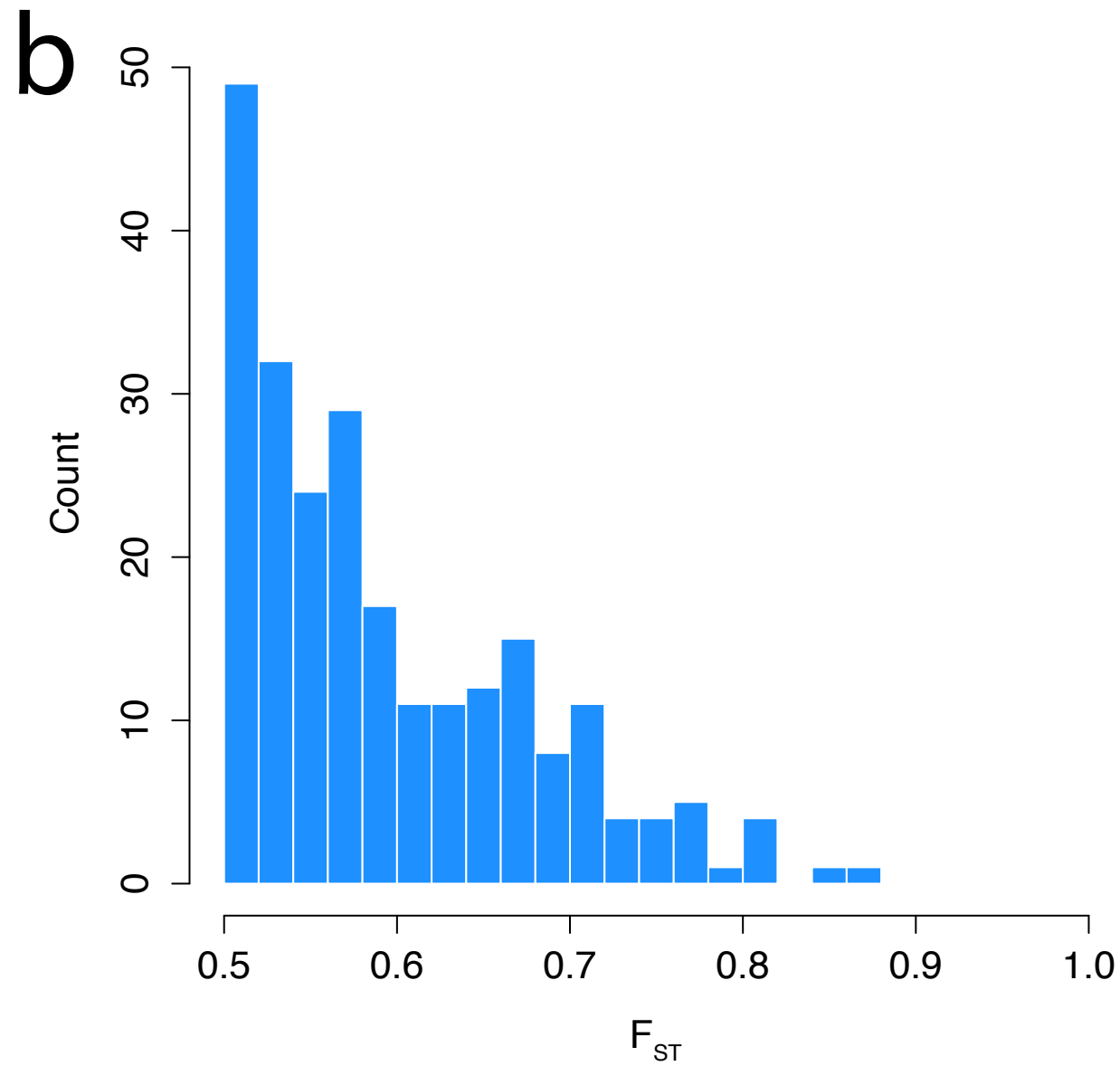

Supplement: S1 Fig — (a) FST distribution for all SNPs genotyped. (b) Distribution of SNPs with FST greater than 0.5. Average FST is weighted by allele frequency and was calculated according to equation 10 in Weir and Cockerham (1984) [20]. (PDF) [file pone.0133292.s001.pdf]

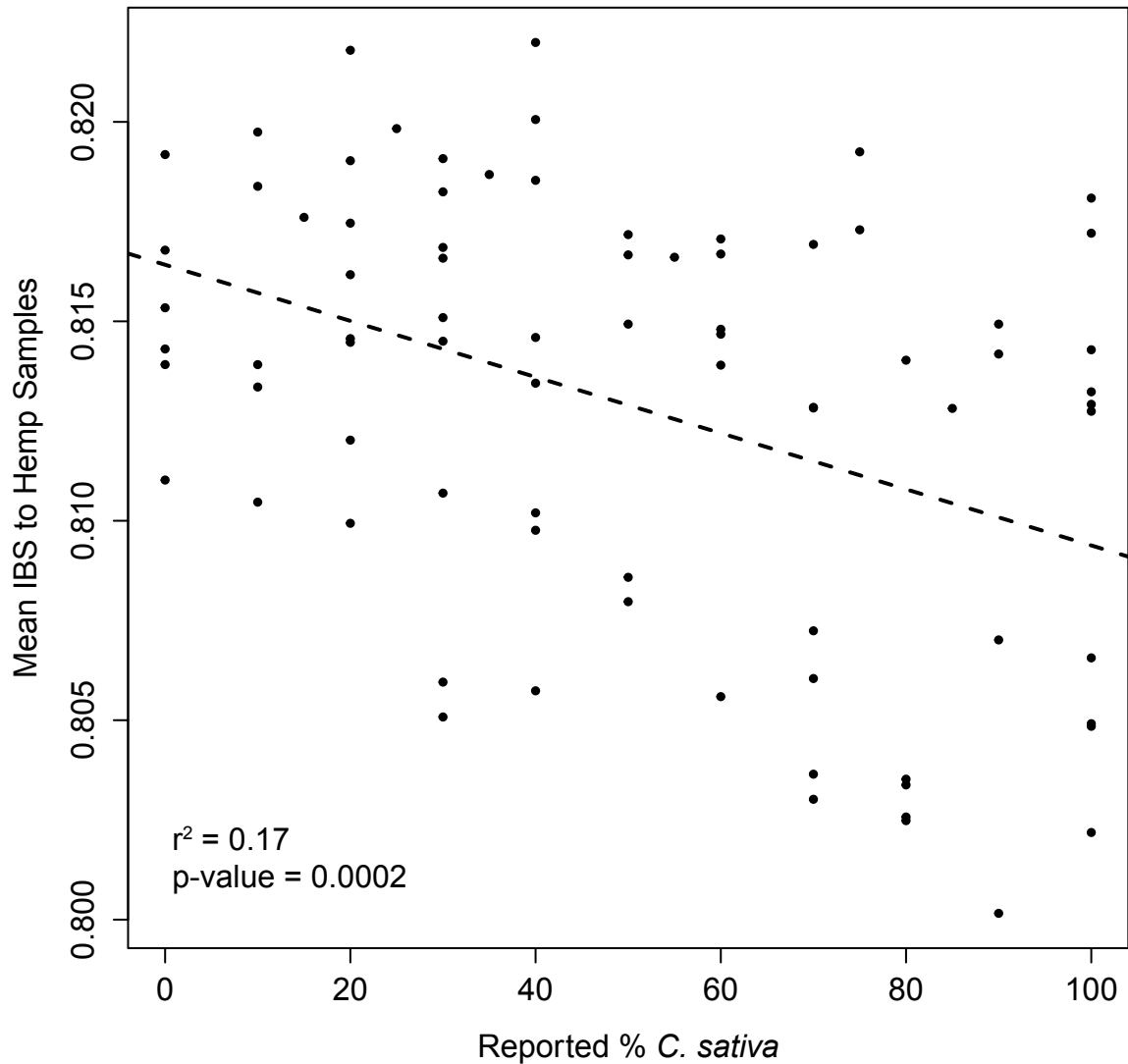

Supplement: S2 Fig — (PDF) [file pone.0133292.s002.pdf]
